# Editorial: Impact of oral and gut microbiome on health and diseases

**DOI:** 10.3389/fcimb.2026.1792511

**Published:** 2026-02-06

**Authors:** Subhadeep Das, Jonathan B. Clayton, Soumyadev Sarkar

**Affiliations:** 1 Department of Biotechnology, School of Life Science & Biotechnology, Adamas University, Kolkata, India; 2 Department of Biology, University of Nebraska at Omaha, Omaha, NE, United States; 3 Nebraska Food for Health Center, University of Nebraska-Lincoln, Lincoln, NE, United States; 4 Department of Food Science and Technology, University of Nebraska-Lincoln, Lincoln, NE, United States; 5 Department of Pathology and Microbiology, University of Nebraska Medical Center, Omaha, NE, United States; 6 Center for Fundamental and Applied Microbiomics, Biodesign Institute, Arizona State University, Tempe, AZ, United States

**Keywords:** disease, dysbiosis, frailty, gut microbiome, health, microplastics, oral microbiome, women’s health

The gut microbiome (10^13^ to 10^14^ cells) and oral microbiome (10^9^ to 10^10^ cells) are significant components of the human microbiome. A healthy microbiome promotes the overall well-being of the individual, whereas dysbiosis leads to various health concerns. The gut and the oral microbiome often communicate through the ‘oral-gut axis’ route. It is important to explore these cross-talks and identify the relevant microbial communities. The goal of this Research Topic is to uncover how oral and gut microbiota, and their crosstalk, regulate human health, drive disease through dysbiosis, and can be precisely modulated for therapeutic benefit. This Research Topic will be an invaluable resource for researchers interested in (1) microbial dysbiosis and disease progression, (2) the influence of diet and lifestyle on oral-gut microbiome composition, and (3) the therapeutic potential of microbiome modulation (**Figure 1**).

**Figure 1:**
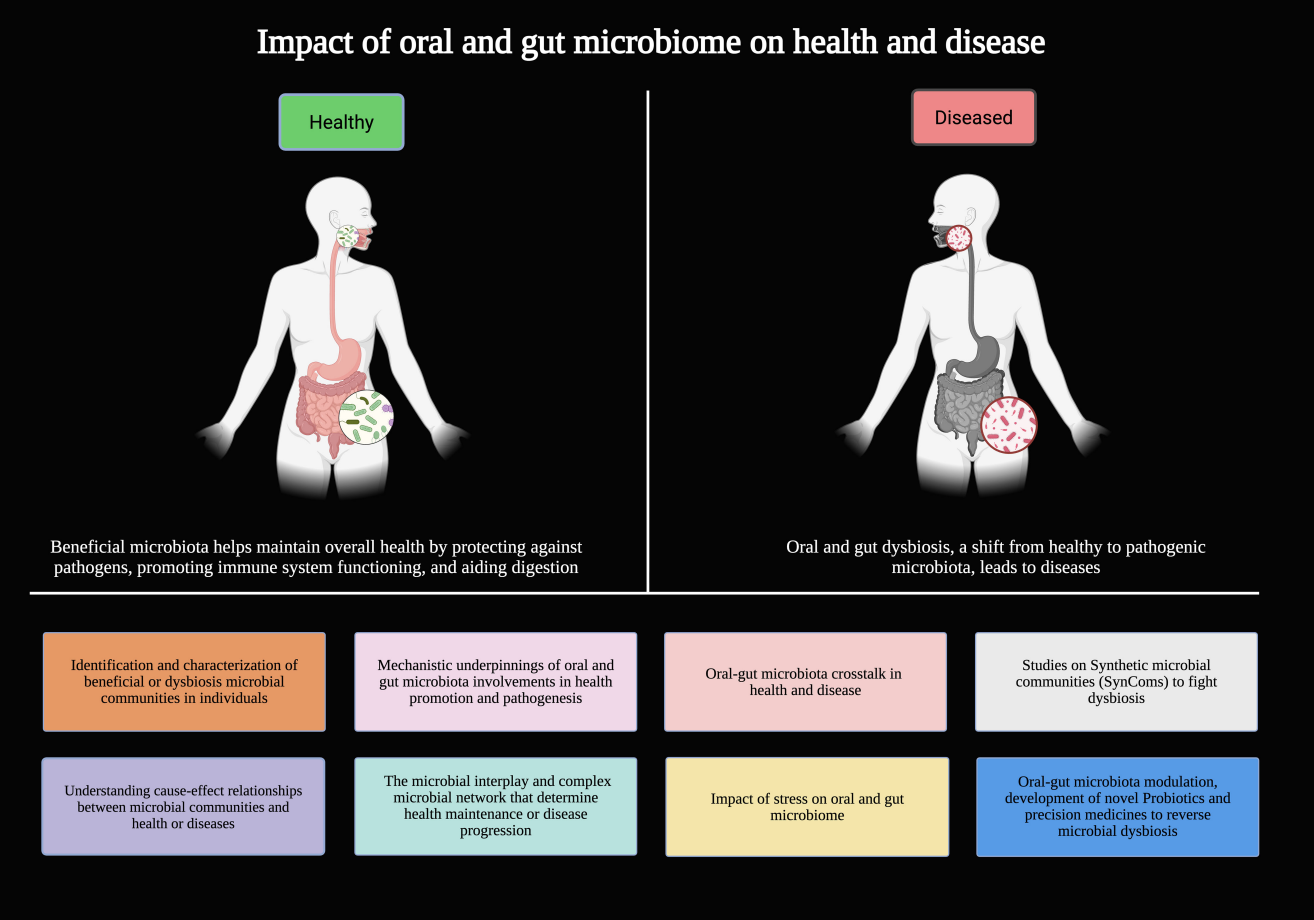
The themes of the Research Topic.

## Microbial dysbiosis and disease progression

A brief report by Barb et al., identified the alterations in oral-gut microbial interplay and compositional overlapping during the first month of inpatient abstinence in twenty-two participants with Alcohol Use Disorder (AUD). These findings indicate a dynamic shift of microbiome populations following alcohol cessation, possibly indicating early stabilization of oral and gastrointestinal environments along with the restoration of site-specific microbial niches. The research highlights the significance of microbial patterns across different sites in the wider context of AUD recovery. The research article from Xia et al. explored the presence of occult gastrointestinal bleeding (GIB), highlighting it as a clinically significant complication of acute ischemic stroke (AIS). Occult GIB occurred in almost 14% of AIS patients and was linked to poorer short- and long-term functional outcomes along with rates of survival. The study also highlighted that the particular enrichment of gut pathogens (*Enterococcus*, *Staphylococcus*, and *Pseudomonas*) during the acute phase has been demonstrated to be independently associated with occult GIB. Gan et al. reviewed the interplay between the oral-gut axis as a novel avenue for comprehending and addressing chronic inflammatory and metabolic disorders. The research investigation conducted by Wang et al. examined the impact of psychological factors on the comorbidity of dental caries and obesity in adolescents via the oral-gut-brain axis. The results indicate that oral and gut microbial populations are integral to disease progression, with abnormalities potentially influencing mental health via the oral-gut-brain axis. Zheng et al. explored the role of *Fusobacterium nucleatum* in ulcerative colitis (UC) and its mechanisms. *Fusobacterium* finds its way through the oral-gut axis, and this study offers new insights into the complex relationship between the oral microbiome and intestinal disease, and offers therapeutic potential. The review from Ren et al. identifying the role of *Streptococcus oralis* residing as a part of the oral cavity is quite intriguing. Otherwise considered a member of the normal flora, it is known to behave as an opportunistic pathogen and cause infectious diseases like infective endocarditis, bloodstream infections, meningitis, ocular infections, and deep-seated abscesses. The review provides a detailed overview of the events associated with *Streptococcus oralis* infection. Another study by Wang et al. demonstrated that the severity of renal fibrosis is associated with intestinal dysbiosis and alterations in serum metabolites. The group subsequently identified biomarkers at various stages of the disease to promote targeted therapeutic interventions. In a similar study, Huang et al. suggested that dysbiosis of the oral microbiota may be associated with the development of metabolism-associated fatty liver disease (MAFLD). This review aimed to validate the relationship between oral microbial diversity and the development of MAFLD, and there is an important association between the diversity of oral microbiota composition and MAFLD. Shi et al. reported on the role of oral microbiota among 38 individuals with major depressive disorder (MDD) along with sleep disturbances, in comparison to 30 healthy volunteers. The group discovered that the relative levels of *Haemophilus* spp. were significantly increased in MDD patients experiencing sleep disturbances. Additionally, the glial fibrillary acidic protein acted as a mediator, influencing the connection between the levels of oral *Haemophilus* spp. and sleep disturbances among individuals with MDD.

The research investigation by Bai et al., explored the differential effects of a high-fat diet (HFD) on salivary and gut microbiome. Using a mouse model and 16s rRNA amplicon sequencing analysis, the group revealed that the distribution of oral and gut microbiota and metabolic pathways was very different in the HFD group. The study concluded that HFD may cause obesity through mechanisms involving pathways, including pentose phosphate, bacterial invasion of epithelial cells, and steroid biosynthesis pathways. Yin-deficiency constitution (YinDC) refers to a traditional Chinese medicine concept characterized by an imbalance state that includes an imbalance in the gut microbiota, resulting from a relative deficiency of Yin fluids within the body. However, it is an established fact that the structures and functions of gut microbiota influence the aging process. The article by Zhai et al. for the very first time, identifies a few significant indicator species influencing YinDC premature aging. The study will help strategize personalized anti-aging strategies. Liu et al. identified essential gut microbial interaction relationships in multiple system atrophy (MSA) utilizing comprehensive multimodal studies. The study revealed consistent results, notably a reduced abundance of *Fusicatenibacter* in MSA. Furthermore, the correlation and network analyses identified distinct interbacterial relationships associated with MSA, notably a novel positive association between the *Ruminococcus gnavus* group and *Erysipelatoclostridium*.

Extensive research indicates that periodontal disease (PD) extends beyond the oral cavity, correlating with the development of diseases in various parts of the body. The research article from the Wang et al. indicates that the periodontitis-associated metabolite isoleucine (Ile) contributes to the development of intestinal barrier dysfunction and inflammatory responses, leading to colitis through the enhancement of NF-κB signaling. Additionally, Ile decreased the expression of ZO-1 and occludin while boosting the levels of pro-inflammatory cytokines (TNF-α, IL-6, and IL-1β) by triggering the NF-κB signaling pathway both *in vivo* and *in vitro*. The research article from Hesami et al. identified the diagnostic potential of periodontal microbes in conjunction with circulating oncomiRNAs for the identification of pancreatic cancer (PC) in a study involving 41 PC patients and 40 age- and sex-matched controls. The research indicated that a higher incidence of the periodontal pathogens *Porphyromonas gingivalis* in females (OR = 2.31; 95% CI 0.98-5.47) and *Aggregatibacter actinomycetemcomitans* in people with diabetes (OR = 3.66; 95% CI 0.47-6.68) correlated with a greater probability of PC. Furthermore, miR-21 and miR-155 have been identified as biomarkers for pancreatic cancer. A separate mini-review by Li et al. pointed out potential associations between PD and prostate ailments, including chronic prostatitis, benign prostatic hyperplasia, and prostate cancer. The investigation examines the pathological mechanisms that govern this link, along with its clinical effects. This article seeks to uncover probable pathogenic mechanisms and provide strategies for the prevention and treatment of prostate conditions through the treatment of PD. Li et al. demonstrated that obstructive sleep apnea (OSA) is linked to specific alterations in salivary microbiota, characterized by decreased microbial diversity and modified functional patterns, potentially leading to early-onset periodontal dysbiosis. *Rothia* has been recognized as a prospective microbial biomarker for periodontitis associated with obstructive sleep apnea (OSA), whereas both *Rothia* and *Parvimonas* may significantly contribute to periodontitis-related OSA. Nonetheless, given that this is only a cross-sectional study, the underlying mechanisms and potential value of microbial biomarkers require validation through long-term studies. The research article by Huang et al. demonstrated that pathogenic periodontal organisms lead to a reduction in gut barrier integrity through immunomodulation of the microbiome. The study stated that *Porphyromonas gingivalis* showed significant pro-inflammatory responses. Li et al. reviewed the cross-talk between PD and chronic kidney disease (CKD), outlining the underlying pathological mechanisms of this relationship. Xi et al. examined how periodontal bacteria may contribute to systemic diseases by altering the gut microbiota via the oral–gut microbiome network, thereby facilitating chronic inflammation.

The oral-gut microbiota axis plays a very important role in the progression of numerous cancers. Gut microbes and metabolites influence the development and progression of breast cancer by regulating the tumor immune response, estrogen metabolism, chemotherapy, and immunotherapy effects. The review by Guo discusses these themes and explores the potential function of the microbiota as a biomarker for prognosis and therapeutic response. Zhang et al. authored a narrative review examining the link between oral microbial variation and biliary tract cancers (BTC). This study examined the roles of bacterial translocation, inflammatory metabolites, and immune modifications in the advancement of cancer progression.

The systematic review conducted by Huang et al. revealed notable alterations in the oral microbiome of individuals with type 2 diabetes mellitus (T2DM) through an analysis of 34 research investigations. Alterations were noted across phylum, genus, and species levels, notably the phylum *Firmicutes*, the genus *Streptococcus*, and the species *Porphyromonas gingivalis*, showing the most noticeable increase. The overall oral bacterial population was typically elevated among individuals with T2DM, whereas bacterial diversity exhibited varied trends across different studies. According to Masoori et al., a study on 500 individuals with diabetes mellitus (DM) from Iran elucidated the prevalence of oral cavity parasites. The study aimed to determine the frequency, socio-economic characteristics, and risk factors of *Entamoeba gingivalis* and *Trichomonas tenax* in DM patients. In one other study by the Deng et al., oral and gut microbiota profiling was done in prediabetes mellitus (Pre-DM) and type 2 diabetes mellitus (T2DM) patients while exploring the association between tongue manifestations and the oral-gut microbiota axis in diabetes progression. The tongue characteristics changed from Pre-DM to T2DM. Approaching techniques like tongue diagnosis with microbiome analysis revealed distinct microbial markers and tongue features, and significantly improved the Pre-DM and T2DM diagnostic capability.

Su et al. demonstrated that the oral-gut microbiota axis has been identified as an essential contributor in the onset of cardiovascular diseases (CVDs). The review emphasized the complex interactions between oral and gut microbiota via microbial translocation, metabolic exchange, and immune signaling, which significantly influence the emergence of various heart conditions. Li et al. examined the salivary and gut microbial signatures of maintenance hemodialysis (MHD) patients suffering from cardiac failure having preserved ejection fraction (HFpEF). The study identified salivary *Anaerocolumna* as a robust biomarker for the HFpEF group.

The review by Yang et al. presented a summary of the positive impact of enterosalivary nitrate metabolism, emphasizing the involvement of oral and gut microbial populations in the enzymatic reduction of nitrate to nitrite. The review examined various nitrate-reduction pathways, factors that regulate nitrate-reducing bacteria, and the interrelationship between systemic health, nitrate consumption, and bacterial distribution. The review article by Zhu et al. elucidated the oral-gut axis as a cohesive basis for comprehending the significance of microbiota in systemic diseases. The study highlighted the necessity for integrated therapeutic techniques that concurrently address both oral and gut ecosystems to reduce systemic as well as local inflammation.

A study by Guo et al. on 25 infected men who have sex with men (MSM), demonstrates significant shifts in the salivary microbiome across different stages of HIV infection, with notable changes in both fungal diversity and functional pathways, notable dominance of *Pseudogymnoascus*, and considerable functional alterations. These findings suggest that the oral microbiome could serve as a valuable diagnostic tool for monitoring HIV-related oral health, with potential implications for therapeutic strategies.

## Impact of fatigue and frailty on oral and gut microbiota

Weakness status in individuals results in deviation from the normal microbiota. Two studies, one of which characterizes the salivary microbiome in healthy individuals under fatigue status. The other explores the gut metagenomics features of frailty. A particular study by Jarmukhanov et al. based on 158 participants from Kazakhstan, showed that microbial diversity decreased significantly with increasing frailty. They proposed a “frailty-associated metabolic signature” in the gut microbiome. This signature suggests multiple interconnected mechanisms through which the microbiome may influence frailty development, including modulation of inflammation, alterations in energy metabolism, and potential impacts on muscle function through microbial metabolites. Another study by Peng et al. on 70 individuals from China demonstrated that the salivary microbiome of fatigued individuals showed decreased diversity and disruption of the microbial community structure, with a significant increase in periodontal disease-related pathogens as compared to that in the control group. They also reported potential cross-talk among the oral microbiome, their related metabolic functions, and the central nervous system across the oral-microbiota-brain axis in the fatigue state.

## Microbial survival strategies

The entry capability of viable but non-cultivable (VBNC) states as a survival strategy under stressful conditions has gained considerable attention in recent years. Due to low metabolic activity, the VNBC cells are highly tolerant of antibiotics and antimicrobials. Ari et al. discuss the VBNC state, its importance in public health, and diagnostic techniques, with a special focus on the VBNC state in oral bacteria.

## Plastics as poisons

Microplastics (MPs), products of the breakdown of large plastics, are omnipresent in terrestrial, marine, and freshwater environments. MPs are known to reach and accumulate in the human gut microbiome through ingestion and inhalation. This disruption has been linked to various health issues, including gastrointestinal disorders, systemic inflammation, and chronic diseases. The gut-brain axis may also be affected, with potential neuroinflammatory consequences. A interesting review by Bora et al. highlights the effects of MPs on human health, emphasizing their impact on the gut microbiome. They discuss the potential connections between MP exposure and cardiometabolic and inflammatory diseases, as well as disorders related to the gut-brain axis.

## Role in pregnancy and women’s health

Two articles discussed the role of the oral and gut microbiome in women’s health and pregnancy. Benslimane et al. showed that the changes in salivary microbiome and biochemical markers were characterized in 45 Qatari pregnant women in their second to third trimester of pregnancy, and there were significant microbial and biochemical shifts during pregnancy. A particular research investigation conducted by Liu et al. has emphasized the crucial role of oral and gut microorganisms in the progression of postmenopausal osteoporosis (PMO), leading to the emergence of the “oral-gut-bone axis” notion. The research involving 21 postmenopausal women, aged 50 to 60, observed notable compositional and functional changes in the oral microbiome linked to bone density health, with specific bacterial species exhibiting substantial intergroup variations.

## Conclusion

The Research Topic significantly advances the understanding of the microbiome of the domain ‘oral-gut axis’. This topic reinforces the concept that oral and gut microbiomes are not separate entities; rather intertwined, having profound effects on host health. There are several discussions of the oral-gut microbiome in the context of diseases and fatigue, such as PD, MAFLD, CKD, and CVD, etc. The topic not only raises the health concerns associated with the gut and oral microbiome but also proposes solutions by proposing the gut-oral microbiome as a biomarker and a therapeutic target in modern medicine.

